# Social support, adherence to Mediterranean diet and physical activity in adults: results from a community-based cross-sectional study

**DOI:** 10.1017/jns.2020.46

**Published:** 2020-11-11

**Authors:** Elpiniki Laiou, Iro Rapti, Georgios Markozannes, Luisella Cianferotti, Lena Fleig, Lisa Marie Warner, Lourdes Ribas, Joy Ngo, Sergio Salvatore, Antonia Trichopoulou, Antonella Vigilanza, Stavroula Tsiara, Georgia Martimianaki, Barbara Pampaloni, Luis Serra Majem, Ralf Schwarzer, Maria Luisa Brandi, Evangelia E. Ntzani

**Affiliations:** 1 Department of Hygiene and Epidemiology, University of Ioannina School of Medicine, Ioannina, Greece; 2 Department of Nursing, University of Ioannina, Ioannina, Greece; 3 Department of Surgery and Translational Medicine, University of Florence, Florence, Italy; 4 Department of Health Psychology, Freie Universität Berlin, Berlin, Germany; 5 MSB Medical School Berlin, Berlin, Germany; 6 Nutrition Research Foundation, Barcelona Science Park, Barcelona, Spain; 7 ISBEM, University of Salento, Lecce, Italy; 8 Hellenic Health Foundation, Athens, Greece; 9 Fondazione F.I.R.M.O., Florence, Italy; 10 University of Las Palmas de Gran Canaria, Research Institute of Biomedical and Health Sciences (IUIBS), Preventive Medicine Service, Centro Hospitalario Universitario Insular Materno Infantil (CHUIMI), Canarian Health Service, Las Palmas, Spain; 11 CIBER de la Fisiopatología de la Obesidad y Nutrición (CIBEROBN), Instituto de Salud Carlos III (ISCIII), Madrid, Spain; 12 Department of Clinical, Health and Rehabilitation Psychology, SWPS University of Social Sciences and Humanities, Wroclaw, Poland; 13 Center for Evidence Synthesis in Health, Department of Health Services, Policy and Practice, School of Public Health, Brown University, Providence, RI, USA; 14 Institute of Biosciences, University Research Center of loannina, University of Ioannina, Ioannina, Greece

**Keywords:** Social support, Mediterranean diet, Physical activity, Cross-sectional

## Abstract

There is a growing recognition that social support can potentially exert consistent or opposing effects in influencing health behaviours. The present paper presents a cross-sectional study, including 2,064 adults from Italy, Spain and Greece, who were participants in a multi-centre randomised controlled trial (C4H study), aiming to examine whether social support is correlated with adherence to a healthy Mediterranean diet and physical activity. Social support data were available for 1,572 participants. The majority of the sample reported emotional support availability (84·5 %), financial support availability (72·6 %) and having one or more close friends (78·2 %). Mediterranean diet adherence was significantly associated with emotional support (*P* = 0·009) and social network support (*P* = 0·021). No statistically significant associations were found between participant physical activity and the social support aspects studied. In conclusion, emotional and social network support may be associated with increased adherence to the Mediterranean diet. However, further research is needed to evaluate the role of social support in adherence to healthy Mediterranean diet.

## Introduction

Social support is defined as the perception that one is accepted, cared for, has assistance available from other people, and that one is part of a supportive social network. It has a complex, multi-level character that involves voluntary associations as well as formal (healthcare professionals and organisations) and informal (family members, friends and peers) relationships with others. Moreover, it is perceived differently on the basis of the recipient's gender, racial or ethnic background, or cultural practices^(1–4)^. Several behaviour change theories, such as the Social Cognitive Theory^(5)^, the Theory of Planned Behaviour^(6)^, the Social-Ecological Model^(7)^ and the Health Action Process Approach^(8)^, draw attention to the importance of social support and social connectedness in achieving and maintaining behaviour change. There is a considerable amount of published research assessing the proposed associations between social support and aspects of physical and mental health such as dietary behaviours, physical activity, smoking, substance or alcohol use, chronic illness management, suicide or self-injury, cardiovascular disease (CVD) and cancer progression. However, in previous research, social support has not been deemed as a consistent predictor of dietary behaviours^(9–11)^, while it appeared to have a moderate effect on exercise behaviours by indirectly improving engagement, compliance and adherence to physical activity^(12)^. Moreover, whether social support is positively or negatively associated with health outcomes and health behaviours depends on the operationalisation of the construct. Perceived social support (prospective availability) and social network size usually show positive associations, while received social support (retrospective assessment of help received) is often found to relate negatively to health and health behaviours^(13,14)^.

Non-communicable diseases, including diabetes and obesity, are a major challenge for health and development. Based on the present evidence, regular physical activity and Mediterranean diet have been proven to help prevent and treat non-communicable diseases, including CVD, type 2 diabetes, some cancers and cognitive impairment^(15–18)^. It also helps to prevent hypertension, overweight and obesity and can improve mental health, quality of life and well-being^(18)^. Recently, there has been an overall aim of providing national and regional level policymakers with guidance on the frequency, duration, intensity, type and total amount of physical activity and the healthy diet needed for the prevention of non-communicable diseases^(19,20)^.

In addition, online social networks have seen enormous growth in popularity in recent years, and yet, there are many uncertainties as to whether, and how, they might be harnessed to improve health behaviours also through the mediation of social support^(21)^. Due to the radical shift in society involving digital technology, the conclusions from the pre-digital era need to be re-evaluated. Focusing on aspects of social support such as emotional support, financial support and social network ties, the aim of the present study was to examine whether there is an association between social support and adherence to a healthy Mediterranean diet and physical activity.

## Methods

### Study design and participants

This cross-sectional study used baseline data from the CREDITS4HEALTH (C4H) study, a parallel-group, multi-centre, randomised controlled community-based trial (RCT). The C4H study aimed to assess the effects of an online platform that supports people in enhancing their level of physical activity and adopting a healthy Mediterranean diet by means of a person-centred approach, providing personalised plans and suggestions along with psychological and social support^(22)^.

Participants were adults, aged 18–65 years, in apparently good health, with the ability of regular internet access through technological devices (PC, tablet and smartphone), and residing in the Florence metropolitan area and the Salento region (Italy), Girona (Spain), and the Pylos Nestoras and Kalamata municipalities (Greece). Individuals with pregnancy, serious weight loss for unexplained reasons (>5 kg in the previous 6 months), chronic disease or disability, presence of conditions with special diet needs and/or advised not to perform physical activity (e.g. end-stage renal disease), inability to use a computer and navigate the web and inability to adhere to the required study centre visits were excluded.

During a baseline visit at the research centres, each participant answered questions related to social background, chronic diseases and pharmacological treatments. Anthropometric measurements (weight, height, BMI, waist circumference and body composition) and blood pressure measurements were recorded by trained staff in accordance with the C4H operations protocol. Participants were also invited to undergo blood testing at associated laboratories for the following parameters: total cholesterol, HDL, LDL, triglycerides, glucose, HbA1c and uric acid.

### Social support assessment

Social support was evaluated using the social support questionnaire (SSQ) of the 2005 National Health and Nutrition Examination Survey (NHANES), a seven-item, self-administered questionnaire assessing the perceived availability of social support and the individuals’ level of satisfaction with the support provided and received. The questions were selected from the Yale Health and Aging Study (MacArthur Studies of Successful Aging)^(23)^ and the Social Network Index – Alameda County Study^(24)^. It includes questions considering the availability of emotional support, the people providing emotional support (friends, spouse, children, co-workers, neighbours and other) as well as the level of received and desired emotional support^(25)^ (Table 2). It should be noted that currently, there is no perfect measure of social support, especially given that the lack of consensus regarding the definition and conceptualisation of social support, as well as the relative gap of strong psychometric evidence in the literature for many of the currently available measures. The SSQ is easily accessible via the Internet, scoring is simple and the questionnaire is quite short, limiting the amount of time consumed to be filled in. Furthermore, the seven included questions are simply formed with comprehensive probes and examples to promote the level of understanding. A very important aspect of the SSQ is the inclusion of questions evaluating social support provided by religious services (e.g. church) and tangible support (financial assistance, material goods or services).

### Physical activity and Mediterranean diet adherence assessment

Physical activity levels were assessed using the General Practice Physical Activity Questionnaire (GPPAQ), a seven-item screening tool developed by the London School of Hygiene & Tropical Medicine as a validated short measure of physical activity used to assess adult (16–74 years) physical activity levels. It includes questions about the type and amount of physical activity involved in one's occupation, hours spent on physical exercise, cycling, walking, housework/childcare and gardening/DIY, providing a four-level categorisation of respondents as active, moderately active, moderately inactive and inactive^(26)^. Considering psychometric properties of the GPPAQ, for reliability, 56 % (70/126) and 67 % (87/129) of controls scored the same at 3 and 12 months, respectively, as they scored at baseline. GPPAQ had 19 % (13/69) sensitivity and 85 % (186/220) specificity^(27)^.

Adherence to the Mediterranean dietary pattern was evaluated using the Mediterranean Diet Adherence Screener (MEDAS), adapted from a previously validated 14-item index^(28)^ with higher scores indicating a healthier diet. MEDAS is a brief 14-item questionnaire which is less time-demanding, less expensive and requires less collaboration from participants than the usual full-length questionnaires or other more comprehensive methods. In addition, it provides the unique window of opportunity to provide feedback to the participant immediately after the questionnaire is completed^(29,30)^. The MEDAS score correlated significantly with the corresponding FFQ PREDIMED score (*r*  0·52; intra-class correlation coefficient 0·51) and in the anticipated directions with the dietary intake reported on the FFQ^(27)^.

To facilitate data analyses, the number of close friends was re-coded into four categories to identify distinct sizes of network: 0; 1–4; 5–9 and 10 or more close friends, and church attendance was re-coded as never, occasionally, weekly and more than weekly, categorisations previously used by McKenzie *et al*.^(31)^. Body mass index (BMI) was re-coded to underweight, normal, overweight and obese based on the WHO classification^(32)^.

### Statistical analysis

Descriptive statistics were calculated to investigate sample characteristics and the distribution of the studied variables. In order to provide more detailed information, baseline characteristics are presented according to recruiting centre as means ± sd, median (IQR) for non-normally distributed data and number (%), using Pearson's *χ*^2^ tests, Fisher's exact tests and Kruskal–Wallis rank tests, as appropriate. The distribution of data was determined using the Shapiro–Wilks test of normality^(32)^. Mixed-effects models were applied to correlate physical activity and Mediterranean diet adherence from the social support variables and calculate effect estimates (Odds ratios (ORs) and *β*). Research centre area was used as a random-effect variable to account for differences across the four research centres. For each area, multiple ordinal and linear regression analyses were conducted to predict physical activity and Mediterranean diet adherence correspondingly from the social support variables. Age and sex were included as covariates in all the models. Complete cases analysis was used with regards to missing data. Differences were considered statistically significant if the *P*-value was <0·05. The present study represents a cross-sectional assessment of the baseline data of the main study; thus, a power analysis was not pertinent. All analyses were performed using Stata (version 13.1; StataCorp, College Station, TX, USA).

Prior to the commencement of the study, ethical approval was obtained by the corresponding competent legal local Ethics Committees of each country and signed informed consent was obtained from all the participants. All the data were collected in compliance with the Declaration of Helsinki.

## Results

Of the 2,064 subjects (361 from Florence, 372 from Salento, 713 from Girona and 618 from Kalamata) recruited from October 2015 to January 2016, social support data were available for 1,572 participants that were included in the present study. Table 1 shows the characteristics of the population under study. The average age was 40·9 ± 11·5 years and 952 (60·6 %) women were included. More than one-third (35·4 %) of the participants had university level education, while 31 % had high school level education. Approximately 42 % of the participants were married or cohabiting, while 32·2 % were single or divorced. The majority of the participants (80·2 %) reported that they were non-smokers and approximately half (52·5 %) reported no alcohol consumption.

**Table 1.** Baseline characteristics of the included C4H study participants

	Florence (*n* 337)	Salento (*n* 319)	Girona (*n* 629)	Pylos-Kalamata (*n* 287)	Total (*N* 1572)	*P*-value*
Age (years)	45 (38–52)	44 (36–52)	40 (32–48)	36 (23–47)	42 (32–50)	<0·001
Men, *n* (%)	133 (39·5)	142 (44·5)	240 (38·2)	105 (36·6)	620 (39·4)	0·183
Marital status, *n* (%)						<0·001
Single or divorced	141 (41·8)	119 (37·3)	131 (20·8)	115 (40·1)	506 (32·2)	
Married or cohabiting	191 (56·7)	195 (61·1)	219 (34·8)	47 (16·4)	652 (41·5)	
Widow/Widower	4 (1·2)	5 (1·6)	4 (0·6)	1 (0·3)	14 (0·9)	
Missing	1 (0·3)	0 (0·0)	275 (43·7)	124 (43·2)	400 (25·4)	
Born in study country, *n* (%)						0·184
Yes	311 (92·3)	306 (95·9)	321 (51·0)	149 (51·9)	1087 (69·1)	
No	25 (7·4)	13 (4·1)	24 (3·8)	14 (4·9)	76 (4·8)	
Missing	1 (0·3)	0 (0·0)	284 (45·2)	124 (43·2)	409 (26·0)	
Education level, *n* (%)						<0·001
University	173 (51·3)	120 (37·6)	197 (31·3)	66 (23·0)	556 (35·4)	
High school	144 (42·7)	141 (44·2)	106 (16·9)	97 (33·8)	488 (31·0)	
Primary/Middle school	18 (5·3)	58 (18·2)	48 (7·6)	0 (0·0)	124 (7·9)	
Missing	2 (0·6)	0 (0·0)	278 (33·2)	124 (43·2)	404 (25·7)	
Smoking, *n* (%)						
Yes	63 (18·7)	76 (23·8)	112 (17·8)	60 (20·9)	311 (19·8)	0·150
No	274 (81·3)	243 (76·2)	517 (82·2)	227 (79·1)	1261 (80·2)	
Alcohol, *n* (%)						
Yes	190 (56·4)	58 (18·2)	476 (75·7)	22 (7·7)	746 (47·5)	<0·001
No	147 (43·6)	261 (81·8)	153 (24·3)	275 (92·3)	826 (52·5)	
BMI (kg/m^2^)	24·2 (21·9–27·5)	26·4 (23·5–29·4)	24·6 (22·2–27·9)	26·1 (22·5–29·2)	25·1 (22·5–28·5)	<0·001
Type 2 diabetes, *n* (%)						
Yes	2 (0·6)	0 (0·0)	6 (1·0)	11 (3·8)	19 (1·2)	<0·001
No	335 (99·4)	319 (100·0)	623 (99·0)	276 (96·2)	1553 (98·8)	
Hypertension, *n* (%)						
Yes	34 (10·1)	36 (11·3)	59 (9·4)	18 (6·3)	147 (9·4)	0·184
No	303 (89·1)	283 (88·7)	570 (90·6)	269 (93·7)	1425 (90·6)	
Number of long-term medications used^a^	0 (0–1)	1 (1–1)	0 (0–1)	0 (0–1)	0 (0–1)^a^	<0·001
Antihypertensive, *n* (%)	24 (7·1)	32 (10·0)	30 (4·8)	13 (4·5)	99 (6·3)	0·008
Hypolipidemic, *n* (%)	8 (2·4)	6 (1·9)	21 (3·3)	8 (2·8)	43 (2·7)	0·593
Physical activity, *n* (%)						<0·001
Active	139 (41·2)	111 (34·8)	334 (53·1)	114 (39·7)	698 (44·4)	
Moderately active	81 (24·0)	64 (20·1)	130 (20·7)	48 (16·7)	323 (20·6)	
Moderately inactive	40 (11·9)	62 (19·4)	59 (9·4)	55 (19·2)	216 (13·7)	
Inactive	77 (22·8)	82 (25·7)	106 (16·8)	70 (24·4)	335 (21·3)	
MEDAS score	8 (6–9)	7 (6–9)	8 (6–9)	6 (5–8)	7 (6–9)	<0·001

Data are presented as median (IQR) unless otherwise indicated.

Hypolipidemic medications included statins and fibrates.

BMI, Body mass index.

a Long-term medication use *N* 1,022, MEDAS score *N* 1,569.

* *P*-value for global comparisons between study areas, evaluated on the non-missing data. Pearson's *χ*^2^ test and Fisher's exact test for categorical variables and Kruskal–Wallis rank test for continuous variables.

At the centre level, the population under study showed statistically significant differences for participant age, marital status, level of education, alcohol consumption, BMI, type 2 diabetes status, number of long-term medications used, physical activity and Mediterranean diet adherence (Table 1). There were no statistically significant differences between study centres with regards to participant sex, country of birth, smoking status, weight and hypertension status. More specifically, the 337 participants from Florence, Italy, had an average age of 45 (38–52) years and 204 (60·5 %) were women. The vast majority of the participants (94·1 %) had a high school or university education. Approximately 57 % of the participants were married or cohabiting, while 42 % were single or divorced. The majority of the participants (81·3 %) reported that they were non-smokers and approximately half (56·4 %) reported alcohol consumption. Most of the participants were active or moderately active (65·2 %) with a median MEDAS score of 8 (6–9). Prevalence of type 2 diabetes and hypertension was low (0·6 % and 10·1 %, respectively). Similar were the results for the participants from Salento, Italy (*n* 319) regarding the average age of 44 (36–52) years, female gender (56·5 %), marital and educational status. Most of the participants were non-smokers (76·2 %) and non-alcohol users (81·8 %). Almost half of the participants were moderately active or active (54·9 %) with a median MEDAS score of 7 (6–9). Only 36 (11·3 %) participants reported hypertension and none was diabetic. Girona, Spain, contributed the largest number of participants (*n* 629). The average age was 40 (32–48) years with a predominance of female gender (64·8 %). Regarding marital status, 34·8 % of the participants were married or cohabiting, 20·8 % were single or divorced, while 43·7 % of the information was missing. Almost half of the participants reported a high school or university education. The majority did not smoke (82·2 %) and consumed alcohol (75·7 %). Overall, 464 (73·8 %) participants were moderately active or active, with median MEDAS score of 8 (6–9). Prevalence of type 2 diabetes and hypertension was low (1 % and 9·4 %, respectively). Lastly, 287 participants came from Pylos-Kalamata, Greece, with a comparatively younger average age of 36 (23–47) and a female gender predominance (63·4 %). In contrast to the other countries, a lower percentage of participants (16·4 %) were married or cohabiting, 40·1 % were single or divorced, while an accountable percentage of information (43·2 %) was missing. Educational level was in accordance with the other centres. The majority neither smoked (79·1 %) nor consumed alcohol (92·3 %). Overall, almost half of the participants (56·4 %) were moderately active or active, with the lowest median MEDAS score of 6 (5–8) compared to the rest of the three centres. Prevalence of type 2 diabetes and hypertension was 3·8 % and 6·3 %, respectively.

The baseline social support characteristics of the C4H participants are presented in Table 2. The majority of the sample reported emotional support availability (84·5 %) or no need for it (4·4 %), 72·6 % reported financial support availability and 78·2 % reported having one or more close friends. Conversely, 59·4 % reported never attending religious services. Statistically significant differences were found between the research centre areas pertaining to emotional support, financial support and religious services attendance, while there were no statistically significant differences pertaining to the participant number of close friends.

**Table 2.** Social support characteristics of the C4H study participants

	Florence	Salento	Girona	Pylos-Kalamata	Total	*P*-value*
Emotional support availability, *n* (%)	*n* 337	*n* 319	*n* 629	*n* 287	*N* 1,572	0·001
Yes	275 (81·6)	265 (83·1)	555 (88·2)	233 (81·2)	1328 (84·5)	
No	29 (8·6)	22 (6·9)	33 (5·2)	15 (5·2)	99 (6·3)	
Do not need help	8 (2·4)	17 (5·3)	23 (3·7)	21 (7·32)	69 (4·4)	
Refused/Do not know	25 (7·4)	15 (4·7)	18 (2·9)	18 (6·3)	76 (4·8)	
More emotional support needed during last 12 months, *n* (%)	*n* 336	*n* 318	*n* 628	*n* 285	*N* 1,567	<0·001
Yes	107 (31·9)	94 (29·6)	192 (30·6)	97 (34·0)	490 (31·3)	
No	133 (39·6)	150 (47·2)	271 (43·2)	150 (52·6)	704 (44·9)	
Refused/Do not know	96 (28·6)	74 (23·3)	165(26·3)	38 (13·3)	373 (23·8)	
Emotional support needed, *n* (%)	*n* 336	*n* 318	*n* 627	*n* 285	*N* 1,566	0·029
A lot more	24 (7·1)	23 (7·2)	45 (7·2)	21 (7·4)	113 (7·2)	
Some more	46 (13·7)	40 (12·6)	102(16·3)	26 (9·1)	214 (13·7)	
A little more	101 (30·1)	93 (29·3)	192 (30·6)	116 (40·7)	502 (32·1)	
Refused/Do not know	165 (49·1)	162(50·9)	288 (45·9)	122 (42·8)	737 (47·1)	
Religious services attendance, *n* (%)	*n* 337	*n* 319	*n* 629	*n* 287	*N* 1,572	<0·001
Never	189 (56·1)	101 (31·7)	537 (85·4)	107 (37·3)	934 (59·4)	
Occasionally	97 (28·8)	126 (39·5)	55 (8·7)	142 (49·5)	420 (26·7)	
Weekly	23 (6·8)	51 (16·0)	9 (1·4)	13 (4·5)	96 (6·1)	
More than weekly	7 (2·1)	29 (9·1)	3 (0·5)	7 (2·4)	46 (2·9)	
Refused/Do not know	21 (6·2)	12 (3·8)	25 (4·0)	18 (6·3)	76 (4·8)	
Religious services attendance per year, median (IQR)	*n* 312	*n* 304	*n* 590	*n* 268	*N* 1,474	<0·001
	0 (0–5)	7·5 (0–52)	0 (0–0)	4 (0–10)	0 (0–6)	
Financial help availability, *n* (%)	*n* 336	*n* 318	*n* 627	*n* 284	*N* 1565	<0·001
Yes	236 (70·2)	215 (67·6)	497 (79·3)	188 (66·2)	1,136 (72·6)	
No	34 (10·1)	40 (12·6)	56 (8·9)	41 (14·4)	171 (10·9)	
Offered but declined	16 (4·8)	17 (5·4)	16 (2·6)	7 (2·5)	56 (3·6)	
Refused/Do not know	50 (14·9)	46 (14·5)	58 (9·3)	48 (16·9)	202 (12·9)	
Number of close friends, *n* (%)	*n* 336	*n* 319	*n* 626	*n* 286	*N* 1,567	0·120
0	34 (10·1)	35 (11·0)	67 (10·7)	34 (11·9)	170 (10·8)	
1–4	156 (46·4)	145 (45·4)	320 (51·1)	143 (50)	764 (48·8)	
5–9	83 (24·7)	81 (25·4)	154 (24·6)	64 (22·4)	382 (24·4)	
10+	27 (8·0)	20 (6·3)	25 (4·0)	7 (2·4)	79 (5·0)	
Refused/Do not know	36 (10·7)	38 (11·9)	60 (9·6)	38 (13·3)	172 (11·0)	
Number of close friends, median (IQR)	*N* 300	*N* 281	*N* 566	*N* 248	*N* 1,395	0·071
	3 (2–5)	3 (2–5)	3 (2–5)	3 (2–5)	3 (2–5)	

* *P*-value for global comparisons between study areas, evaluated on the non-missing data. Pearson's *χ*^2^ test for categorical variables and Kruskal–Wallis rank test for continuous variables.

The results of the mixed effect models analyses are presented in Table 3 (physical activity) and Table 4 (Mediterranean diet adherence). Mediterranean diet adherence was significantly associated with emotional support availability (*P* = 0·009) and social network support in terms of the number of close friends (*P* = 0·021), while there were no statistically significant differences found with regards to emotional support needs, financial support availability and religious services attendance. No statistically significant associations were found between participant physical activity (categorised in four levels) and any of the social support aspects studied. The unadjusted results of the mixed effect models (Supplementary Tables S1 and S2) and the results of the multivariate analyses for each research area (Supplementary Tables S3–S10) are included in the Supplementary material.

**Table 3.** Baseline associations between social support and physical activity (GPPAQ)

Question	Answer	Summary OR^a^	95 % CI	*P*-value	*N*	Overall *P*-value
Can you count on anyone to provide you with emotional support such as talking over problems or helping you make a difficult decision?	No^b^	1			1,496	0·252
	Yes	0·93	0·63–1·37	0·713		
	I do not need help	1·41	0·76–2·60	0·276		
In the last 12 months, could you have used more emotional support than you received?	Yes^b^	1			1,194	0·812
	No	1·03	0·83–1·27	0·812		
Emotional support: Would you say that you could have used…	A lot more^b^	1			829	0·100
	Some	1·36	0·89–2·07	0·159		
	A little more	1·51	1·03–2·21	0·033		
How often do you attend church or religious services?	Never^b^	1			1,489	0·609
	Some	0·94	0·75–1·18	0·609		
How often do you attend church or religious services?	Never^b^	1			1,496	0·274
	Occasionally	1·01	0·79–1·28	0·951		
	Weekly	0·77	0·52–1·15	0·198		
	More than weekly	0·65	0·37–1·14	0·135		
If you need some extra help financially, could you count on anyone to help you?	No^b^	1			1,363	0·239
	Yes	0·77	0·56–1·05	0·096		
	Would not accept it	0·73	0·41–1·29	0·282		
In general, how many close friends do you have?	0^b^	1			1,395	0·772
	1–4	1·12	0·82–1·52	0·470		
	5–9	1·12	0·80–1·57	0·515		
	10+	1·30	0·79–2·15	0·303		

a OR provided from the performed ordinal regression and adjusted for age and sex with area specified as a random factor (i.e. random intercept models).

b Reference category.

**Table 4.** Baseline associations between social support and Mediterranean diet adherence (MEDAS)

Question	Answer	*β*^a^	95 % CI	*P*-value	*N*	Overall *P*-value
Can you count on anyone to provide you with emotional support such as talking over problems or helping you make a difficult decision?	No^b^	0			1,493	0·009
	Yes	0·67	0·21–1·12	0·004		
	I do not need help	0·90	0·21–1·58	0·010		
In the last 12 months, could you have used more emotional support than you received?	Yes^b^	0			1,191	0·655
	No	0·06	−0·20 to 0·32	0·655		
Emotional support: Would you say that you could have used…	A lot more^b^	0			826	0·590
	Some	0·17	−0·35 to 0·69	0·523		
	A little more	−0·02	−0·49 to 0·44	0·930		
How often do you attend church or religious services?	Never^b^	0			1,486	0·104
	Some	−0·22	−0·49 to 0·5	0·104		
How often do you attend church or religious services?	Never^b^	0			1,493	0·404
	Occasionally	−0·23	−0·51 to 0·06	0·119		
	Weekly	−0·26	−0·75 to 0·23	0·306		
	More than weekly	−0·23	−0·92 to 0·46	0·509		
If you need some extra help financially, could you count on anyone to help you?	No^b^	0			1,360	0·930
	Yes	0·05	−0·31 to 0·42	0·778		
	Would not accept it	0·13	−0·56 to 0·81	0·719		
In general, how many close friends do you have?	0^b^	0			1,392	0·021
	1–4	0·26	−0·11 to 0·63	0·169		
	5–9	0·46	0·06–0·86	0·024		
	10+	0·82	0·23–1·41	0·006		

a Adjusted for age and sex with area specified as a random factor (i.e. random intercept models).

b Reference category.

No statistically significant differences were found between the participants with available social support data and the participants with no available social support data in terms of sex and BMI, while the group of the participants with missing social support data was statistically significantly younger (35·1 ± 13·6 years *v*. 40·9 ± 11·5 years; *P* < 0·001).

## Discussion

Our work assessed the correlation of social support parameters with adherence to a healthy Mediterranean diet and physical activity in 1,572 healthy participants from three Mediterranean countries. Mediterranean diet adherence was significantly associated with emotional support availability and social network support. That is, participants who did not report a lack of emotional support and those who reported a higher number of friends achieved higher Mediterranean adherence scores. In contrast, no statistically significant associations were found between Mediterranean diet adherence and emotional support need, religious service attendance and financial support. On the other hand, none of the social support aspects (emotional social support, financial support availability, social network availability and religious service attendance) were statistically significantly associated with physical activity level.

### Comparison to previous studies

With regards to the available research on the relationship between social support and physical activity, the available evidence is limited in size and in methodological rigour mainly comprised of cross-sectional studies^(33,34)^. Overall, the evidence on adults is inconclusive ranging from positive associations in earlier studies^(35)^ to no or unclear evidence^(34,36–39)^. Most studies adopted a cross-sectional design, they involved mainly healthy participants, they focused on the social support provided by friends and family, while a combination of self-reported and objectively measured level of physical activity was used to assess physical activity. Interestingly, provision of social support has been related to small, but significant associations with physical activity in children and adolescents^(34,40)^; cross-sectional and longitudinal studies have shown that social support provided by parents, father, mother, friends and relatives is positively and consistently related to higher physical activity levels in adolescents^(40)^. Given that self-efficacy and competence mediate the relationship between social support and physical activity, it is possible that social support indirectly influences physical activity levels through self-efficacy and other possible mediating constructs (e.g. enjoyment). Moreover, it is likely that the ways in which parents and friends provide social support and influence activity levels are different. For example, friends might contribute to positive experiences in physical education or organised physical activities, while parents could create a foundation for lifelong habits in physical activity in their children at an early age and provide support for their ongoing participation in physical activity during adolescence. Further research might investigate these possible mechanisms in more detail^(40)^.

Similarly to our study which significantly associated Mediterranean diet adherence to emotional support availability and social network support, previous research on the associations between social support and diet showed that the inclusion of planning for social support/social change has been associated with enhanced effectiveness of dietary interventions, resulting in increases in fruit and vegetable intakes^(9–11,41–43)^. Nevertheless, there is a lack of evidence considering the Mediterranean diet exclusively. Socialisation in the form of sitting around the table and sharing food in company of family and friends (conviviality), a form of social support, is considered an inherent aspect of Mediterranean diet lifestyle^(44)^. Yet, the role of the Mediterranean diet as a holistic lifestyle pattern, and not exclusively as a healthier diet, needs further evaluation. Perhaps the relationship to health may exist for the overall Mediterranean diet as a healthy lifestyle pattern, and not necessarily for an isolated aspect of its components.

### Strengths and limitations

Strengths of the present study include its size and the inclusive sample of participants in apparent good health from multiple research centres in three Mediterranean countries, a population that has not been previously studied extensively in terms of associations between social support, Mediterranean diet adherence and physical activity. Correspondingly, to our knowledge, this is one of the first studies to investigate religious services attendance in association with Mediterranean diet and physical activity among Mediterranean populations. Moreover, the present study represents one of the few efforts to investigate the relationship between social support and Mediterranean diet exclusively, as one of the well-established healthy dietary patterns.

The present study is also subject to certain limitations that should be considered. In cross-sectional studies, causal relationships cannot be established between physical activity and social support. Thus, it is not possible to rule out the presence of reverse causality in the results found, that is, people who received more social support are those who were already active. The possibility that this relationship is bidirectional must also be considered, that is, that inactive people who receive social support also become more active or maintain their physical activity levels^(41)^. Moreover, dietary data on Mediterranean diet are based on self-reports, which on top of the cross-sectional study design may limit the relevance of the conclusions. Another possible limitation is that the content validity of some questionnaire items might have been reduced due to the translation of items into Italian, Spanish and Greek. However, all questionnaires were translated from English to all study languages and were checked by bilingual native speakers to ensure that the items correctly captured each construct. Moreover, another limitation of the present study is related to the secondary analyses. The percentage of missing income responses in social support, Mediterranean diet and physical activity questionnaires may impact the association between social support and Mediterranean diet or physical activity. There is also the potential for recall bias. Furthermore, due to a technical error, data were missing for a number of participants. While the missing sample was of a somewhat younger average age, no statistically significant differences were found between the participants with available social support data and the participants with no available social support data in terms of sex and BMI. Moreover, taking into consideration the fact that the study included population from three Mediterranean countries, the use of information in the non-Mediterranean population may be limited.

Overall, methodological inconsistencies exist within the literature on the associations between social support and behavioural change. Social support has been measured using various tools; however, in many cases, these scales were modified for use, or authors use non-validated, custom scales to measure social support. There is a wide number of measures used to appraise social integration or support in the general population or specific patient groups (e.g. arthritis, cancer, CVD and diabetes)^(41)^. Nonetheless, currently, there is no perfect measure of social support, given the lack of consensus regarding the definition and conceptualisation of social support, as well as the relative gap of strong psychometric evidence in the literature for many of the available measures^(45)^. This is problematic because this lack of consistency could lead to imprecise measurement, which has been previously recognised as a challenge^(40)^.

### Implications for future research and practice

Further research is needed to investigate the effects of social support on physical activity and Mediterranean diet adherence including valid measures of social support and its individual components and detailed reporting of potential effect modifiers such as conviviality. Future research should also include the assessment of interventions targeting social support and their impact on the adoption of a healthier lifestyle. Furthermore, while the present study found no association between religious service attendance and Mediterranean diet adherence or physical activity, different religious beliefs and practices may have a different impact on health-related behaviours; the identification of specific factors contributing to the effect of religious participation on health behaviours requires further investigation. Finally, the emergence of digital social networking is quickly altering traditional social contacts. Further research is needed to investigate the potential of digital social networking in altering the effect of social support on health behaviours.

Nowadays, the promotion of healthy diet, healthy weight control and physical activity maintenance are considered key parts in forming a sustainable public health policy toward better population health and the prevention of diseases such as CVD, type 2 diabetes and some cancers. Recognising potential social support mediators for use in evidence-based interventions is a crucial step in the improvement of the interventions aiming to encourage individuals and families in making dietary and physical activity choices that may meaningfully improve the health of present and future generations.

## Conclusions

Our findings suggest that emotional support availability and number of close friends may be positively associated with Mediterranean diet adherence. However, traditional social contacts may not be strongly associated neither with physical activity, nor with healthy diet adherence. The potential of digital technologies in altering the effect of social support on health behaviours needs further investigation. To that end, the further conceptualisation of social support and development of valid and up to date social support measures are needed.

## Supplementary material

For supplementary material accompanying this paper visit https://doi.org/10.1017/jns.2020.46. click here to view supplementary material
