# Supplementary material for: Social support, adherence to Mediterranean diet and physical activity in adults: results from a community-based cross-sectional study
Source: J Nutr Sci. 2020 Nov 11;9:e53. doi: 10.1017/jns.2020.46 (PMC7681167; doi:10.1017/jns.2020.46)
Supplement: Supplementary file 1 [file S2048679020000464sup001.docx]

**Table S1.** Unadjusted baseline associations between social support and physical activity (GPPAQ)

| **Question** | **Answer** | **Summary OR^†^** | **95% CI** | **P-value** | **N** | **Overall**  **P-value** |
| --- | --- | --- | --- | --- | --- | --- |
| Can you count on anyone to provide you with emotional support such as talking over problems or helping you make a difficult decision? | No* | 1 |  |  | 1496 | 0.022 |
|  | Yes | 0.97 | 0.66 – 1.43 | 0.890 |  |  |
|  | I don't need help | 1.92 | 1.05 – 3.49 | 0.033 |  |  |
| In the last 12 months, could you have used more emotional support than you received? | Yes* | 1 |  |  | 1194 | 0.671 |
|  | No | 1.05 | 0.85 – 1.29 | 0.671 |  |  |
| Emotional support: Would you say that you could have used… | A lot more* | 1 |  |  | 829 | 0.100 |
|  | Some | 1.40 | 0.93 – 2.13 | 0.111 |  |  |
|  | A little more | 1.50 | 1.04 – 2.19 | 0.032 |  |  |
| How often do you attend church or religious services? | Never* | 1 |  |  | 1489 | 0.082 |
|  | Some | 0.82 | 0.66 – 1.03 | 0.082 |  |  |
| How often do you attend church or religious services? | Never* | 1 |  |  | 1496 | 0.057 |
|  | Occasionally | 0.88 | 0.70 – 1.12 | 0.308 |  |  |
|  | Weekly | 0.66 | 0.44 – 0.98 | 0.041 |  |  |
|  | More than weekly | 0.55 | 0.31 – 0.95 | 0.034 |  |  |
| If you need some extra help financially, could you count on anyone to help you? | No* | 1 |  |  | 1363 | 0.502 |
|  | Yes | 0.84 | 0.63 – 1.14 | 0.272 |  |  |
|  | Wouldn't accept it | 0.96 | 0.55 – 1.68 | 0.882 |  |  |
| In general, how many close friends do you have? | 0* | 1 |  |  | 1395 | 0.786 |
|  | 1 to 4 | 1.15 | 0.85 – 1.56 | 0.368 |  |  |
|  | 5 to 9 | 1.17 | 0.84 – 1.64 | 0.342 |  |  |
|  | 10+ | 1.21 | 0.74 – 1.98 | 0.458 |  |  |

**†** Unadjusted with area specified as a random factor (i.e. random intercept models).

*Reference category

**Table S2.** Unadjusted baseline associations between social support and Mediterranean Diet adherence (MEDAS)

| **Question** | **Answer** | **beta^†^** | **95% CI** | **P-value** | **N** | **Overall**  **P-value** |
| --- | --- | --- | --- | --- | --- | --- |
| Can you count on anyone to provide you with emotional support such as talking over problems or helping you make a difficult decision? | No* | 0 |  |  | 1493 | 0.041 |
|  | Yes | 0.57 | 0.12– 1.03 | 0.014 |  |  |
|  | I don't need help | 0.69 | 0.00 – 1.37 | 0.050 |  |  |
| In the last 12 months, could you have used more emotional support than you received? | Yes* | 0 |  |  | 1191 | 0.511 |
|  | No | 0.09 | -0.17 – 0.35 | 0.511 |  |  |
| Emotional support: Would you say that you could have used… | A lot more* | 0 |  |  | 826 | 0.727 |
|  | Some | 0.16 | -0.36 – 0.68 | 0.551 |  |  |
|  | A little more | 0.02 | -0.45 – 0.48 | 0.944 |  |  |
| How often do you attend church or religious services? | Never* | 0 |  |  | 1486 | 0.367 |
|  | Some | -0.12 | -0.39 – 0.14 | 0.367 |  |  |
| How often do you attend church or religious services? | Never* | 0 |  |  | 1493 | 0.814 |
|  | Occasionally | -0.13 | -0.42 – 0.15 | 0.361 |  |  |
|  | Weekly | -0.14 | -0.63– 0.36 | 0.586 |  |  |
|  | More than weekly | -0.11 | -0.80 – 0.58 | 0.761 |  |  |
| If you need some extra help financially, could you count on anyone to help you? | No* | 0 |  |  | 1360 | 0.884 |
|  | Yes | -0.09 | -0.45 – 0.27 | 0.632 |  |  |
|  | Wouldn't accept it | -0.12 | -0.80 – 0.56 | 0.733 |  |  |
| In general, how many close friends do you have? | 0* | 0 |  |  | 1392 | 0.024 |
|  | 1 to 4 | 0.20 | -0.18 – 0.57 | 0.282 |  |  |
|  | 5 to 9 | 0.39 | 0.01 – 0.80 | 0.055 |  |  |
|  | 10+ | 0.84 | 0.24 – 1.44 | 0.006 |  |  |

**†** Unadjusted with area specified as a random factor (i.e. random intercept models).

*Reference category

**Table S3.** Baseline associations between social support and physical activity (GPPAQ) - Florence

| **Question** | **Answer** | **Summary OR^†^** | **95% CI** | **P-value** | **N** | **Overall**  **P-value** |
| --- | --- | --- | --- | --- | --- | --- |
| Can you count on anyone to provide you with emotional support such as talking over problems or helping you make a difficult decision? | No* | 1 |  |  | 312 | 0.433 |
|  | Yes | 1.00 | 0.49 – 2.02 | 0.994 |  |  |
|  | I don't need help | 2.99 | 0.50 – 17.77 | 0.229 |  |  |
| In the last 12 months, could you have used more emotional support than you received? | Yes* | 1 |  |  | 240 | 0.349 |
|  | No | 1.26 | 0.78 – 2.02 | 0.349 |  |  |
| Emotional support: Would you say that you could have used… | A lot more* | 1 |  |  | 171 | 0.009 |
|  | Some | 0.80 | 0.32 – 1.98 | 0.629 |  |  |
|  | A little more | 2.16 | 0.96 – 4.88 | 0.064 |  |  |
| How often do you attend church or religious services? | Never* | 1 |  |  | 315 | 0.321 |
|  | Some | 1.25 | 0.81 – 1.92 | 0.321 |  |  |
| How often do you attend church or religious services? | Never* | 1 |  |  | 316 | 0.189 |
|  | Occasionally | 1.43 | 0.89 – 2.29 | 0.135 |  |  |
|  | Weekly | 0.63 | 0.28 – 1.41 | 0.261 |  |  |
|  | More than weekly | 1.74 | 0.44 – 6.91 | 0.429 |  |  |
| If you need some extra help financially, could you count on anyone to help you? | No* | 1 |  |  | 286 | 0.991 |
|  | Yes | 1.03 | 0.53 – 2.01 | 0.923 |  |  |
|  | Wouldn't accept it | 1.08 | 0.35 – 3.36 | 0.894 |  |  |
| In general, how many close friends do you have? | 0* | 1 |  |  | 300 | 0.797 |
|  | 1 to 4 | 0.84 | 0.43 – 1.66 | 0.621 |  |  |
|  | 5 to 9 | 1.08 | 0.52 – 2.27 | 0.836 |  |  |
|  | 10+ | 0.93 | 0.35 – 2.43 | 0.881 |  |  |

**†** Adjusted for age and sex.

*Reference category

**Table S4.** Baseline associations between social support and Mediterranean Diet adherence (MEDAS) – Florence

| **Question** | **Answer** | **beta^†^** | **95% CI** | **P-value** | **N** | **Overall**  **P-value** |
| --- | --- | --- | --- | --- | --- | --- |
| Can you count on anyone to provide you with emotional support such as talking over problems or helping you make a difficult decision? | No* | 0 |  |  | 311 | 0.064 |
|  | Yes | 0.82 | 0.03– 1.61 | 0.043 |  |  |
|  | I don't need help | 1.64 | -0.00 – 3.29 | 0.050 |  |  |
| In the last 12 months, could you have used more emotional support than you received? | Yes* | 0 |  |  | 239 | 0.854 |
|  | No | 0.05 | -0.48 – 0.58 | 0.854 |  |  |
| Emotional support: Would you say that you could have used… | A lot more* | 0 |  |  | 170 | 0.954 |
|  | Some | 0.04 | -1.01 – 1.10 | 0.934 |  |  |
|  | A little more | -0.07 | -1.03 – 0.89 | 0.885 |  |  |
| How often do you attend church or religious services? | Never* | 0 |  |  | 314 | 0.372 |
|  | Some | -0.22 | -0.71 – 0.26 | 0.372 |  |  |
| How often do you attend church or religious services? | Never* | 0 |  |  | 315 | 0.676 |
|  | Occasionally | -0.25 | -0.78 – 0.27 | 0.342 |  |  |
|  | Weekly | -0.06 | -0.97 – 0.86 | 0.905 |  |  |
|  | More than weekly | -0.78 | -2.50 – 0.94 | 0.370 |  |  |
| If you need some extra help financially, could you count on anyone to help you? | No* | 0 |  |  | 285 | 0.418 |
|  | Yes | 0.43 | -0.33 – 1.18 | 0.267 |  |  |
|  | Wouldn't accept it | -0.01 | -1.25 – 1.22 | 0.982 |  |  |
| In general, how many close friends do you have? | 0* | 0 |  |  | 299 | 0.266 |
|  | 1 to 4 | 0.21 | -0.56 – 0.98 | 0.595 |  |  |
|  | 5 to 9 | 0.38 | -0.45 – 1.21 | 0.372 |  |  |
|  | 10+ | 0.98 | -0.08 – 2.04 | 0.069 |  |  |

**†** Adjusted for age and sex.

*Reference category

**Table S5.** Baseline associations between social support and physical activity (GPPAQ) - Salento

| **Question** | **Answer** | **Summary OR^†^** | **95% CI** | **P-value** | **N** | **Overall**  **P-value** |
| --- | --- | --- | --- | --- | --- | --- |
| Can you count on anyone to provide you with emotional support such as talking over problems or helping you make a difficult decision? | No* | 1 |  |  | 304 | 0.901 |
|  | Yes | 0.93 | 0.39 – 2.20 | 0.862 |  |  |
|  | I don't need help | 1.15 | 0.32 – 4.12 | 0.831 |  |  |
| In the last 12 months, could you have used more emotional support than you received? | Yes* | 1 |  |  | 244 | 0.365 |
|  | No | 0.80 | 0.49 – 1.30 | 0.365 |  |  |
| Emotional support: Would you say that you could have used… | A lot more* | 1 |  |  | 156 | 0.284 |
|  | Some | 2.15 | 0.82 – 5.63 | 0.119 |  |  |
|  | A little more | 1.50 | 0.63 – 3.56 | 0.360 |  |  |
| How often do you attend church or religious services? | Never* | 1 |  |  | 306 | 0.683 |
|  | Some | 0.91 | 0.58 – 1.43 | 0.683 |  |  |
| How often do you attend church or religious services? | Never* | 1 |  |  | 307 | 0.174 |
|  | Occasionally | 1.06 | 0.65 – 1.72 | 0.826 |  |  |
|  | Weekly | 0.79 | 0.42 – 1.49 | 0.473 |  |  |
|  | More than weekly | 0.46 | 0.21 – 1.01 | 0.054 |  |  |
| If you need some extra help financially, could you count on anyone to help you? | No* | 1 |  |  | 272 | 0.290 |
|  | Yes | 0.59 | 0.30 – 1.14 | 0.116 |  |  |
|  | Wouldn't accept it | 0.60 | 0.21 – 1.73 | 0.344 |  |  |
| In general, how many close friends do you have? | 0* | 1 |  |  | 281 | 0.914 |
|  | 1 to 4 | 1.21 | 0.62 – 2.36 | 0.566 |  |  |
|  | 5 to 9 | 1.16 | 0.56 – 2.40 | 0.682 |  |  |
|  | 10+ | 0.97 | 0.36 – 2.62 | 0.955 |  |  |

**†** Adjusted for age and sex.

*Reference category

**Table S6.** Baseline associations between social support and Mediterranean Diet adherence (MEDAS) – Salento

| **Question** | **Answer** | **beta^†^** | **95% CI** | **P-value** | **N** | **Overall**  **P-value** |
| --- | --- | --- | --- | --- | --- | --- |
| Can you count on anyone to provide you with emotional support such as talking over problems or helping you make a difficult decision? | No* | 0 |  |  | 304 | 0.010 |
|  | Yes | 0.97 | 0.04 – 1.90 | 0.041 |  |  |
|  | I don't need help | 2.11 | 0.75 – 3.48 | 0.003 |  |  |
| In the last 12 months, could you have used more emotional support than you received? | Yes* | 0 |  |  | 244 | 0.412 |
|  | No | -0.24 | -0.81 – 0.33 | 0.412 |  |  |
| Emotional support: Would you say that you could have used… | A lot more* | 0 |  |  | 156 | 0.596 |
|  | Some | 0.58 | -0.55 – 1.70 | 0.313 |  |  |
|  | A little more | 0.41 | -0.60 – 1.42 | 0.424 |  |  |
| How often do you attend church or religious services? | Never* | 0 |  |  | 306 | 0.248 |
|  | Some | -0.31 | -0.83 – 0.21 | 0.248 |  |  |
| How often do you attend church or religious services? | Never* | 0 |  |  | 307 | 0.725 |
|  | Occasionally | -0.30 | -0.87 – 0.27 | 0.295 |  |  |
|  | Weekly | -0.32 | -1.06 – 0.41 | 0.386 |  |  |
|  | More than weekly | -0.25 | -1.16 – 0.66 | 0.589 |  |  |
| If you need some extra help financially, could you count on anyone to help you? | No* | 0 |  |  | 272 | 0.978 |
|  | Yes | 0.08 | -0.68 – 0.84 | 0.836 |  |  |
|  | Wouldn't accept it | 0.05 | -1.22 – 1.32 | 0.937 |  |  |
| In general, how many close friends do you have? | 0* | 0 |  |  | 281 | 0.594 |
|  | 1 to 4 | 0.43 | -0.36 – 1.21 | 0.286 |  |  |
|  | 5 to 9 | 0.58 | -0.26 – 1.42 | 0.177 |  |  |
|  | 10+ | 0.29 | -0.87 – 1.46 | 0.621 |  |  |

**†** Adjusted for age and sex.

*Reference category

**Table S7.** Baseline associations between social support and physical activity (GPPAQ) - Girona

| **Question** | **Answer** | **Summary OR^†^** | **95% CI** | **P-value** | **N** | **Overall**  **P-value** |
| --- | --- | --- | --- | --- | --- | --- |
| Can you count on anyone to provide you with emotional support such as talking over problems or helping you make a difficult decision? | No* | 1 |  |  | 611 | 0.548 |
|  | Yes | 0.83 | 0.42 – 1.65 | 0.589 |  |  |
|  | I don't need help | 1.30 | 0.43 – 4.01 | 0.640 |  |  |
| In the last 12 months, could you have used more emotional support than you received? | Yes* | 1 |  |  | 463 | 0.786 |
|  | No | 0.95 | 0.66 – 1.36 | 0.786 |  |  |
| Emotional support: Would you say that you could have used… | A lot more* | 1 |  |  | 339 | 0.209 |
|  | Some | 1.84 | 0.93 – 3.63 | 0.080 |  |  |
|  | A little more | 1.59 | 0.85 – 2.97 | 0.145 |  |  |
| How often do you attend church or religious services? | Never* | 1 |  |  | 602 | 0.218 |
|  | Some | 0.74 | 0.46 – 1.20 | 0.218 |  |  |
| How often do you attend church or religious services? | Never* | 1 |  |  | 604 | 0.606 |
|  | Occasionally | 0.77 | 0.45 – 1.31 | 0.340 |  |  |
|  | Weekly | 0.56 | 0.18 – 1.71 | 0.310 |  |  |
|  | More than weekly | 0.98 | 0.09 – 11.20 | 0.987 |  |  |
| If you need some extra help financially, could you count on anyone to help you? | No* | 1 |  |  | 569 | 0.765 |
|  | Yes | 0.88 | 0.51 – 1.54 | 0.665 |  |  |
|  | Wouldn't accept it | 0.67 | 0.23 – 1.97 | 0.468 |  |  |
| In general, how many close friends do you have? | 0* | 1 |  |  | 566 | 0.225 |
|  | 1 to 4 | 1.49 | 0.90 – 2.47 | 0.123 |  |  |
|  | 5 to 9 | 1.50 | 0.86 – 2.63 | 0.154 |  |  |
|  | 10+ | 2.50 | 0.98 – 6.39 | 0.055 |  |  |

**†** Adjusted for age and sex.

*Reference category

**Table S8.** Baseline associations between social support and Mediterranean Diet adherence (MEDAS) – Girona

| **Question** | **Answer** | **beta^†^** | **95% CI** | **P-value** | **N** | **Overall**  **P-value** |
| --- | --- | --- | --- | --- | --- | --- |
| Can you count on anyone to provide you with emotional support such as talking over problems or helping you make a difficult decision? | No* | 0 |  |  | 611 | 0.162 |
|  | Yes | 0.76 | -0.07– 1.60 | 0.073 |  |  |
|  | I don't need help | 0.38 | -0.88 – 1.65 | 0.551 |  |  |
| In the last 12 months, could you have used more emotional support than you received? | Yes* | 0 |  |  | 463 | 0.590 |
|  | No | 0.12 | -0.32 – 0.57 | 0.590 |  |  |
| Emotional support: Would you say that you could have used… | A lot more* | 0 |  |  | 339 | 0.745 |
|  | Some | -0.19 | -1.06 – 0.69 | 0.673 |  |  |
|  | A little more | -0.31 | -1.11 – 0.50 | 0.459 |  |  |
| How often do you attend church or religious services? | Never* | 0 |  |  | 602 | 0.558 |
|  | Some | -0.18 | -0.80 – 0.43 | 0.558 |  |  |
| How often do you attend church or religious services? | Never* | 0 |  |  | 604 | 0.135 |
|  | Occasionally | 0.10 | -0.57 – 0.77 | 0.764 |  |  |
|  | Weekly | -1.74 | -3.31 – -0.17 | 0.030 |  |  |
|  | More than weekly | -0.14 | -3.84 – 1.56 | 0.407 |  |  |
| If you need some extra help financially, could you count on anyone to help you? | No* | 0 |  |  | 569 | 0.749 |
|  | Yes | -0.16 | -0.83 – 0.52 | 0.652 |  |  |
|  | Wouldn't accept it | -0.52 | -1.87 – 0.83 | 0.453 |  |  |
| In general, how many close friends do you have? | 0* | 0 |  |  | 566 | 0.164 |
|  | 1 to 4 | 0.26 | -0.38 – 0.90 | 0.423 |  |  |
|  | 5 to 9 | 0.63 | -0.07 – 1.33 | 0.079 |  |  |
|  | 10+ | 0.91 | -0.20 – 2.02 | 0.109 |  |  |

**†** Adjusted for age and sex.

*Reference category

**Table S9.** Baseline associations between social support and physical activity (GPPAQ) – Pylos Kalamata

| **Question** | **Answer** | **Summary OR^†^** | **95% CI** | **P-value** | **N** | **Overall**  **P-value** |
| --- | --- | --- | --- | --- | --- | --- |
| Can you count on anyone to provide you with emotional support such as talking over problems or helping you make a difficult decision? | No* | 1 |  |  | 269 | 0.639 |
|  | Yes | 0.88 | 0.34 – 2.27 | 0.790 |  |  |
|  | I don't need help | 1.33 | 0.39 – 4.56 | 0.655 |  |  |
| In the last 12 months, could you have used more emotional support than you received? | Yes* | 1 |  |  | 247 | 0.201 |
|  | No | 1.36 | 0.85 – 2.16 | 0.201 |  |  |
| Emotional support: Would you say that you could have used… | A lot more* | 1 |  |  | 163 | 0.297 |
|  | Some | 0.69 | 0.24 – 1.97 | 0.489 |  |  |
|  | A little more | 1.24 | 0.52 – 2.96 | 0.625 |  |  |
| How often do you attend church or religious services? | Never* | 1 |  |  | 266 | 0.871 |
|  | Some | 1.04 | 0.66 – 1.64 | 0.871 |  |  |
| How often do you attend church or religious services? | Never* | 1 |  |  | 269 | 0.880 |
|  | Occasionally | 0.98 | 0.62 – 1.57 | 0.941 |  |  |
|  | Weekly | 1.45 | 0.53 – 3.95 | 0.469 |  |  |
|  | More than weekly | 1.26 | 0.26 – 6.13 | 0.778 |  |  |
| If you need some extra help financially, could you count on anyone to help you? | No* | 1 |  |  | 236 | 0.392 |
|  | Yes | 0.65 | 0.35 – 1.21 | 0.175 |  |  |
|  | Wouldn't accept it | 0.80 | 0.16 – 3.98 | 0.781 |  |  |
| In general, how many close friends do you have? | 0* | 1 |  |  | 248 | 0.752 |
|  | 1 to 4 | 0.89 | 0.44 – 1.78 | 0.734 |  |  |
|  | 5 to 9 | 0.72 | 0.34 – 1.56 | 0.412 |  |  |
|  | 10+ | 1.29 | 0.30 – 5.62 | 0.732 |  |  |

**†** Adjusted for age and sex.

*Reference category

**Table S10.** Baseline associations between social support and Mediterranean Diet adherence (MEDAS) – Pylos Kalamata

| **Question** | **Answer** | **beta^†^** | **95% CI** | **P-value** | **N** | **Overall**  **P-value** |
| --- | --- | --- | --- | --- | --- | --- |
| Can you count on anyone to provide you with emotional support such as talking over problems or helping you make a difficult decision? | No* | 0 |  |  | 267 | 0.823 |
|  | Yes | -0.27 | -1.37 – 0.83 | 0.628 |  |  |
|  | I don't need help | -0.44 | -1.83 – 0.95 | 0.535 |  |  |
| In the last 12 months, could you have used more emotional support than you received? | Yes* | 0 |  |  | 245 | 0.106 |
|  | No | 0.44 | -0.09 – 0.98 | 0.106 |  |  |
| Emotional support: Would you say that you could have used… | A lot more* | 0 |  |  | 161 | 0.386 |
|  | Some | 0.88 | -0.38 – 2.15 | 0.169 |  |  |
|  | A little more | 0.46 | -0.57 – 1.48 | 0.380 |  |  |
| How often do you attend church or religious services? | Never* | 0 |  |  | 264 | 0.772 |
|  | Some | 0.08 | -0.46 – 0.61 | 0.772 |  |  |
| How often do you attend church or religious services? | Never* | 0 |  |  | 267 | 0.143 |
|  | Occasionally | -0.08 | -0.62 – 0.46 | 0.766 |  |  |
|  | Weekly | 0.78 | -0.46 – 2.02 | 0.214 |  |  |
|  | More than weekly | 1.52 | -0.12 – 3.16 | 0.069 |  |  |
| If you need some extra help financially, could you count on anyone to help you? | No* | 0 |  |  | 234 | 0.009 |
|  | Yes | 0.06 | -0.67 – 0.79 | 0.867 |  |  |
|  | Wouldn't accept it | 2.57 | 0.85 – 4.29 | 0.004 |  |  |
| In general, how many close friends do you have? | 0* | 0 |  |  | 246 | 0.595 |
|  | 1 to 4 | 0.13 | -0.65– 0.91 | 0.737 |  |  |
|  | 5 to 9 | 0.03 | -0.83 – 0.90 | 0.938 |  |  |
|  | 10+ | 1.13 | -0.57 – 2.83 | 0.191 |  |  |

**†** Adjusted for age and sex.

*Reference category
